# Rapid Paediatric Sequencing (RaPS): comprehensive real-life workflow for rapid diagnosis of critically ill children

**DOI:** 10.1136/jmedgenet-2018-105396

**Published:** 2018-07-26

**Authors:** Lamia Mestek-Boukhibar, Emma Clement, Wendy D Jones, Suzanne Drury, Louise Ocaka, Andrey Gagunashvili, Polona Le Quesne Stabej, Chiara Bacchelli, Nital Jani, Shamima Rahman, Lucy Jenkins, Jane A Hurst, Maria Bitner-Glindzicz, Mark Peters, Philip L Beales, Hywel J Williams

**Affiliations:** 1 GOSgene, Genetics and Genomic Medicine, UCL Great Ormond Street Institute of Child Health, London, UK; 2 Department of Clinical Genetics, North East Thames RegionalGenetics Service, Great Ormond Street Hospital for Children NHS Trust, London, UK; 3 Congenica Ltd, Bioinnovation Data Centre, Wellcome Genome Campus, Cambridge, UK; 4 Genetics and Genomic Medicine, UCL Great Ormond Street Institute of Child Health, London, UK; 5 NE Thames Regional Genetics Laboratory, Great Ormond Street Hospital, London, UK; 6 Respiratory, Critical Care and Anaesthesia Unit, UCL Great Ormond Street Institute of Child Health and Great Ormond Street NHS Foundation Trust, London, UK

**Keywords:** whole genome sequencing, paediatric intensive care unit, rapid diagnosis, genomics, rare disease

## Abstract

**Background:**

Rare genetic conditions are frequent risk factors for, or direct causes of, paediatric intensive care unit (PICU) admission. Such conditions are frequently suspected but unidentified at PICU admission. Compassionate and effective care is greatly assisted by definitive diagnostic information. There is therefore a need to provide a rapid genetic diagnosis to inform clinical management.

To date, whole genome sequencing (WGS) approaches have proved successful in diagnosing a proportion of children with rare diseases, but results may take months to report. Our aim was to develop an end-to-end workflow for the use of rapid WGS for diagnosis in critically ill children in a UK National Health Service (NHS) diagnostic setting.

**Methods:**

We sought to establish a multidisciplinary Rapid Paediatric Sequencing team for case selection, trio WGS, rapid bioinformatics sequence analysis and a phased analysis and reporting system to prioritise genes with a high likelihood of being causal.

**Results:**

Trio WGS in 24 critically ill children led to a molecular diagnosis in 10 (42%) through the identification of causative genetic variants. In 3 of these 10 individuals (30%), the diagnostic result had an immediate impact on the individual’s clinical management. For the last 14 trios, the shortest time taken to reach a provisional diagnosis was 4 days (median 8.5 days).

**Conclusion:**

Rapid WGS can be used to diagnose and inform management of critically ill children within the constraints of an NHS clinical diagnostic setting. We provide a robust workflow that will inform and facilitate the rollout of rapid genome sequencing in the NHS and other healthcare systems globally.

## Introduction

An increasing proportion of critically ill children have one or more chronic diseases that contribute to, or directly precipitate, paediatric intensive care admission.^1^ Rare genetic conditions are present in a significant proportion of elective and emergency admissions. Uncertainty about diagnosis and often prognosis contributes to the difficulty of planning optimal care. Achieving a rapid molecular diagnosis in critically ill children with a rare genetic disease may improve the basis for such plans including informing on the potential value of highly invasive treatments.^2 3^ Reaching a genetic diagnosis also precludes the need for further diagnostic investigations, which may be invasive, painful and expensive.^4^ For the family, a molecular diagnosis enables accurate genetic counselling and ends the diagnostic odyssey.^5^ However, obtaining a genetic diagnosis in a timely manner in critically ill individuals is frequently challenging and often not possible. Factors preventing a rapid genetic diagnosis include heterogeneity of disease, limited availability of broad genetic testing, long time frames involved in standard diagnostic molecular testing and limited knowledge of the molecular basis for most genetic disorders.

Recent advances in genome sequencing and bioinformatics provide a solution to many of the traditional hurdles presented by rare diseases. Whole exome sequencing (WES) approaches, where only the coding sequence of genes is targeted, have proven successful in diagnosing a proportion of children and adults with rare diseases in both the research and diagnostic arenas.^5–10^ Whole genome sequencing (WGS) which, unlike WES, is not biased to particular genomic regions, is now frequently being performed in the research setting and is beginning to be used for diagnostic purposes. A comparison between the two methods has shown that WGS is the preferred option for testing Mendelian disorders.^11^ In the UK, WGS is being extended into the healthcare environment through the 100,000 Genomes Project (100KGP)^12 13^; however, feedback of results is currently expected to take many months. In contrast, recent studies from the USA^14–16^ and the Netherlands^17^ have shown the benefit of rapid WGS in acutely ill children and have clearly demonstrated the cost effectiveness of this technique compared with standard genetic testing.^16^ In these studies, however, a rapid diagnosis was made through the use of modified laboratory equipment and working procedures incompatible with standard diagnostic laboratory practices in the UK or involved the use of a predetermined gene list that was applied to all patients.

The aim of this study was therefore to expand on previous rapid sequencing studies by developing the first end-to-end workflow using rapid WGS to diagnose critically ill children in a National Health Service (NHS) setting. We specifically set out to devise a workflow that used standard laboratory equipment, adhered to the standard working practices of a diagnostic laboratory and performed an unbiased analysis of the whole genome. For this study, rapidity of diagnosis is not the only or even most important issue to address as sustainability of a rapid WGS sequencing service in the context of an NHS diagnostic laboratory is of paramount importance.

Specifically, this workflow begins with the identification of an eligible patient on the paediatric intensive care unit (PICU) and ends with the delivery of a diagnostic report.

To do this, we set up a multidisciplinary team to ensure our workflow seamlessly transitioned between the various specialities. We adopted a fully prospective two-stage approach whereby the first 10 trios were used to iteratively develop a workflow, which was then applied to the next 14 trios.

An essential goal of this study was to develop a workflow integrated within an existing service laboratory that could be adopted by other diagnostic centres. We therefore make this information freely available for others to use.

## Methods

The study was undertaken in an NHS tertiary children’s hospital with a 23-bed multidisciplinary PICU and a 20-bed paediatric cardiac intensive care unit (CICU).

Signed informed parental consent for participation in this study was obtained in all cases.

### Rapid Paediatric Sequencing (RaPS) team

**Figure 1:**
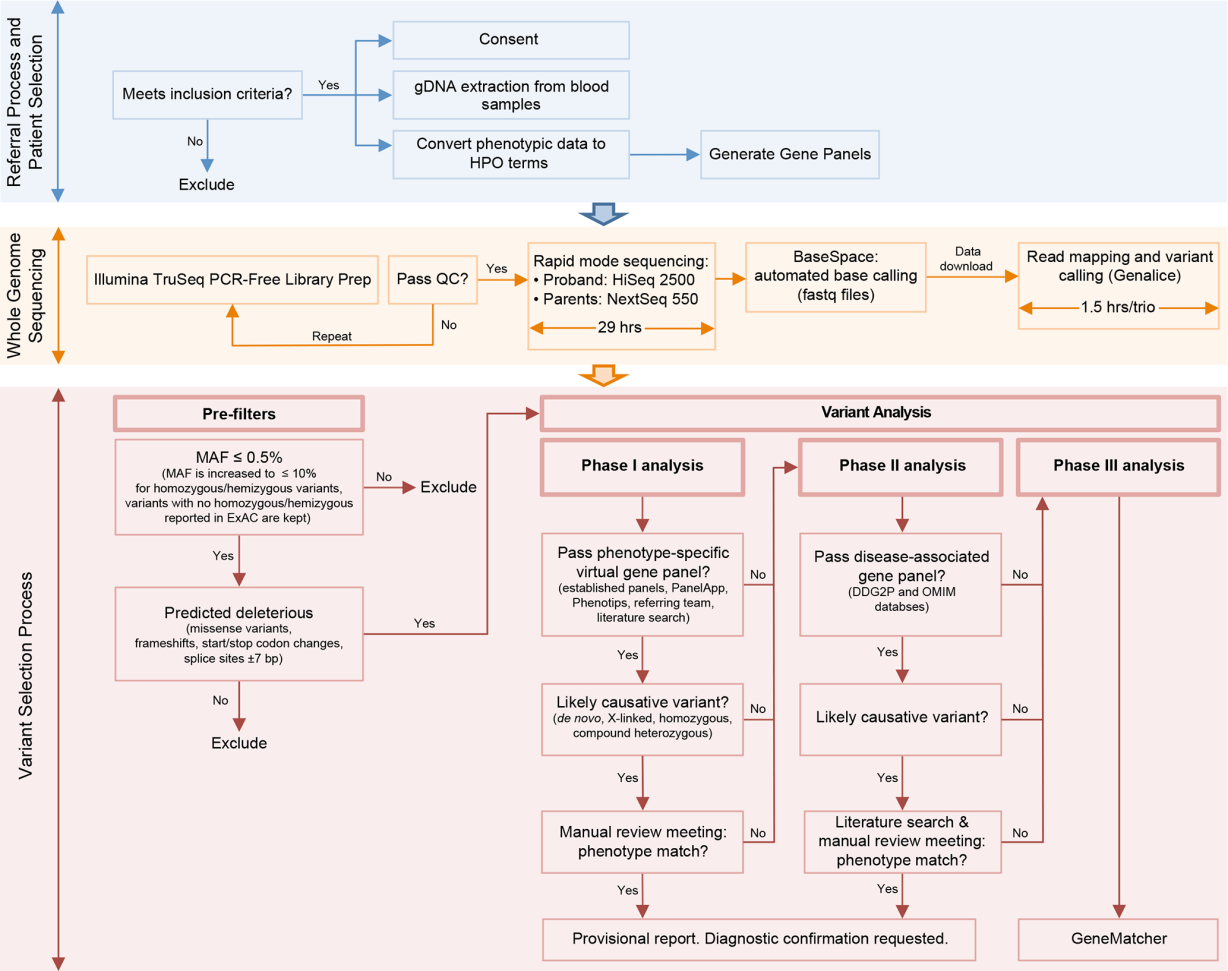
Description of RaPS workflow. A flow diagram representing the three stages of the RaPS workflow showing how trio samples progress from the stage of patient referral to the issuing of a diagnostic report. This diagram provides brief details of the variant filtering steps applied to samples and the phased analysis strategy. Detailed methods are provided in online supplementary material 2.

We established a multidisciplinary RaPS team consisting of clinical geneticists, research and clinical scientists. The RaPS team was supported by PICU clinicians and other paediatric specialist teams who identified critically ill individuals for inclusion. Our workflow comprises detailed inclusion and exclusion criteria, clinical data capture and conversion to Human Phenotype Ontology (HPO) terms, rapid DNA extraction and WGS, a rapid bioinformatics analysis pipeline, tiered reporting of potentially causative variants, multidisciplinary team discussion and validation of results in an accredited NHS diagnostic laboratory (figure 1).

10.1136/jmedgenet-2018-105396.supp1 Supplementary data

### Implementing standard operational procedures: the first 10 cases

The first 10 cases recruited were used to strengthen our standard operational procedures to ensure high quality, consistency and reproducibility throughout the RaPS workflow. First, a rapid bioinformatics pipeline was identified, tested and implemented in the RaPS workflow. Regular meetings were set up with the clinical genetics team and the referring clinicians to ensure: prompt consenting, patient assessment against inclusion and exclusion criteria, updates on clinical information, review of genomic variants and return of clinical findings proceeded in a timely manner. Additionally, close collaboration was put in place between the hospital’s accredited diagnostic laboratory to establish protocols for prioritised gDNA extraction and regular access to sequencing machines. Furthermore, diagnostic confirmation of clinical findings using Sanger sequencing was integrated in the RaPS workflow in collaboration with accredited clinical scientists.

### Inclusion criteria

Suitable participants were clinically ascertained by a specialist physician or PICU consultant between August 2015 and October 2017. The first 10 trios were run as proof-of-principle to establish workflow systems. This allowed us to iteratively review our inclusion criteria and led to the development of the following list that we applied to the remaining 14 trios:

1. Essential inclusion criteria:
  - Trio DNA samples must be available.
  - Parental consent.
  - Suspected underlying monogenic cause.
2. High priority criteria:
  - A genetic diagnosis may significantly alter the clinical management of the patient.
  - The phenotype or family history data strongly implicate a genetic aetiology, but the phenotype does not correspond with a specific disorder for which a genetic test targeting a specific gene is available on a clinical basis.
  - A patient presents with a defined genetic disorder that demonstrates a high degree of genetic heterogeneity, making WGS analysis a more practical approach than a gene panel test.
  - A patient presents with a likely genetic disorder, but specific diagnostic tests available for that phenotype have failed to arrive at a diagnosis or are not accessible within a reasonable timeframe. Such tests may include a gene panel test, microarray, biochemical test, imaging or biopsy.
  - Imminent demise of patient not likely.

In summary, using these criteria we aimed to identify individuals with a high likelihood of having a monogenic disorder and focused on those individuals in whom achieving a genetic diagnosis would be likely to inform clinical management in the acute setting. For excluded individuals who were felt to be at high risk of imminent demise, standard diagnostic testing or access to WGS through the 100KGP^7^ was suggested where appropriate in order to offer an explanation and inform future genetic counselling for their parents and family members.

### Recruitment and consent

To expedite the identification and pathogenic assessment of causative genes, we recruited biological trios consisting of proband and both parents.^15^ Trios were consented by the referring clinician or the clinical genetics team. A template for recording clinical and family history at time of consent was developed in order to standardise data capture and improve workflow. Phenotypic information provided by a clinical geneticist, or specialist paediatrician, was captured as HPO terms to facilitate bespoke gene panel design for each patient (online supplementary table 1).

Participants were given the choice of opting in or out of return of secondary findings as guided by recommendations from the American College of Medical Genetics and Genomics (ACMG).^18^

#### Genomic assays

Detailed methods are found in online supplementary material 1. Briefly, DNA was extracted using Chemagic-STAR (Hamilton, USA). Whole genome gDNA libraries were prepared using TruSeq DNA PCR-Free Library Prep (Illumina, USA) following manufacturer’s advice starting with 1 μg of sheared gDNA. Parental samples were pooled at equimolar concentrations and sequenced on Illumina NextSeq 550 High-Output Mode (29 hours). Patient samples were sequenced on Illumina HighSeq 2500 Dual Flow Cell, Rapid Run Mode (27 hours) except for patient samples from the last two trios, which were sequenced on NextSeq 550 High-Throughput Mode (29 hours). Mapping and variant calling were performed using a Genalice appliance running Genalice Map 2.5.5 including Mapping, Variant Calling and the Population Calling module for trio analysis (Genalice Core BV, Netherlands). Genalice default configuration files were used for WGS mapping and trio variant detection.

#### Variant interpretation

Ingenuity Variant Analysis software (Qiagen, USA) was used to identify rare variants predicted to result in loss of function or to have a functional effect on the protein. Variants with a frequency of ≤0.5% in 1000 Genomes,^19^ Exome Aggregation Consortium (ExAC)^20^ and Exome Variant Server were investigated. Additionally, we also performed a complimentary analysis to identify candidate recessive and X-linked variants with a high carrier frequency. For this analysis, we used a more permissive allele frequency cut-off of ≤10% with those variants with no reported homozygous or hemizygous genotypes in ExAC included for further analysis. We then selected only variants that were predicted to be deleterious (simple nucleotide variants, frameshifts, start/stop codon changes, splice sites ±7bp). Genetic filters were set to investigate autosomal recessive homozygous, autosomal recessive compound heterozygous, X-linked and de novo variants. For variants within genes with a recessive mode of inheritance in the phase I analysis, we also shortlisted predicted deleterious heterozygous variants. When such variants were identified, we manually inspected the genomic region using Integrative Genome Viewer (IGV) software^21^ to detect potential structural variants on the second allele.

Data were analysed in a three-stage process (phases I–III) to prioritise likely causative genes and facilitate prompt return of results (figure 1). All putative pathogenic calls were manually assessed using IGV to ensure they were true variants and not technical artefacts.

### Phase I analysis

In phase I, we restricted the genes analysed to those with a high probability of being implicated in the individual’s disorder. This required analysis of a bespoke phase I gene panel generated from gene lists provided by the referring clinical teams in conjunction with an HPO-derived panel using the following resources: Genomics England PanelApp,^22^ Phenotips^23^ and Online Inheritance in Man (OMIM) Gene Map^24^ (see online supplementary material 2). If a phase I variant was deemed to be causal and explain the entire phenotype, no further analysis of WGS was deemed necessary.

### Phase II analysis

Individuals entered phase II analysis when no likely causative variant was identified in phase I analysis or when a phase I candidate variant did not fully explain the reported phenotype. Phase II comprised a broad analysis of genes known to be associated with developmental disorders and disease more generally. Phase II involved analysis of genes from the Developmental Disorders Genotype-Phenotype database^6^ and OMIM Morbid genes.^24^

### Phase III analysis

Individuals entered phase III analysis if no causal variants were identified from phase I or phase II or their phenotype was not fully explained. The aim of phase III was to open up the analysis to select variants in any gene with compelling evidence for causality based on the deleteriousness of the variant and either animal models, expression pattern or in silico predictions. Where a genetic diagnosis was not achieved in phase I or II, variants of potential research interest from phase III were shared with the online portal GeneMatcher^25^ to identify potential collaborators with variants in the same gene.

#### Multidisciplinary review

Variants identified from phases I and II analysis were triaged by the core RaPS team including a clinical geneticist and research scientists. Any variants deemed to be potentially relevant to the individual’s phenotype were scored according to ACMG variant interpretation guidelines^26^ These were then reviewed in a genomic multidisciplinary team (MDT) meeting comprising at least two clinical geneticists, the referring team when available and clinical and research scientists in order to determine a consensus on pathogenicity and the need for further investigations.

#### Feedback of results

Variants assessed as pathogenic or likely pathogenic and contributing to the individual’s phenotype following MDT discussion were fed back to referring clinicians by the clinical genetics team. At this point, a provisional research results report was generated. Diagnostic results were validated in an accredited laboratory using Sanger sequencing of the full trio. The return of results to the family was led by clinical genetics team or by the referring clinicians with the assistance of clinical genetics team. If no likely pathogenic variants were identified after phase II analysis, a ‘no primary findings’ research results report was issued to the referring clinical team detailing the analysis performed and plan for continued research analysis.

#### Role of funding source

The funding source had no role in the design of the study, the collection, analysis or interpretation of the data, or the writing of the report. Authors LB, EC, WJ, JAH, SR, MB-G and HJW had access to the raw data.

## Results

### RaPS workflow: from patient to variant

**Figure 2:**
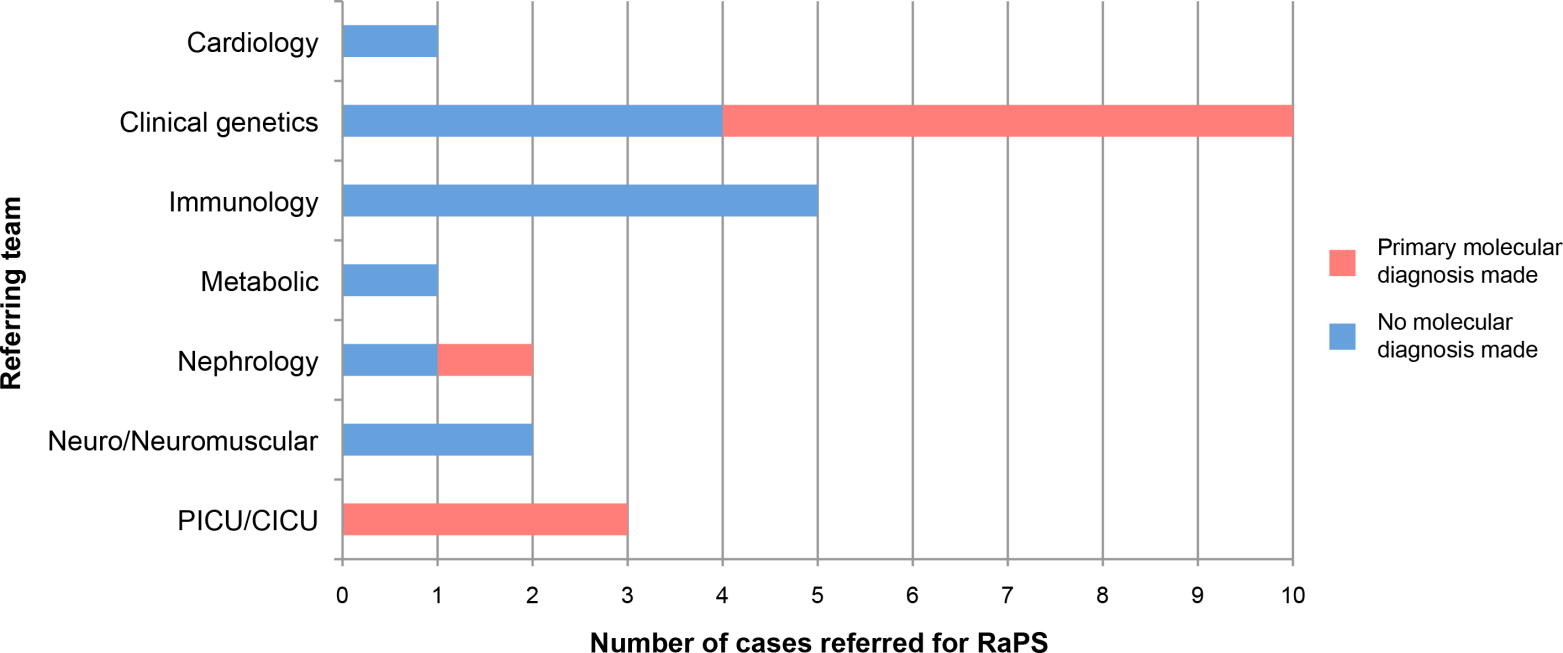
Number of trios referred and diagnoses made per clinical speciality. Graph showing the number of patients referred from specialist clinical teams and whether that patient received a molecular diagnosis.

Individuals recruited were on average known to seven specialist medical teams in the hospital (figure 2 and online supplementary figure 1). Mean age of affected individuals at point of sequencing was 15.86 months (range 7 days–13 years 2 months) with a median age of 2.5 months (online supplementary figure 2). Using our inclusion criteria (developed through analysis of first 10 trios), we recruited 14 of 29 trios (48%) referred to us, with the remaining being excluded for a range of reasons (online supplementary figure 3).

WGS of the 24 trios generated an average of 5.8 million genomic variants per trio (online supplementary table 2), including those seen in only one parent. The time taken for read mapping and variant calling of the sequence data using the Genalice appliance ranged from 10 min to 40 min with an average time of 19 min per sample. The number of variants per workflow stage and phase is indicated in online supplementary figure 4. Our coverage metrics showed that on average 88% of the proband’s genome had at least 10× coverage and an average of 67% of the parent’s genome had at least 10× coverage (online supplementary table 3). Similar coverage rates were obtained for the coding regions investigated during variant interpretation.

A primary molecular diagnosis (classified as a diagnosis accounting for the majority of an individual’s phenotype) was achieved in 10 out of 24 trios (42%) (table 1). Of note, all diagnoses were made in phase I analysis (online supplementary table 4). Diagnostic variants comprised four de novo mutations, three pairs of compound heterozygous variants and three homozygous variants.

**Table 1:** Summary of diagnoses made in the RaPS cohort

RaPS ID	Gene	MIM	Phenotype	Inheritance
Diagnosis made through RaPS				
RaPS_01	*POLE1*	174 762	Facial dysmorphism, immunodeficiency, livedo, short stature (FILS) syndrome	Compound heterozygote
RaPS_02	*COL3A1*	120 180	Ehlers-Danlos syndrome, type IV	De novo
RaPS_05	*CHD7*	608 892	CHARGE syndrome	De novo
RaPS_07	*PIGT*	615 398	Multiple congenital anomalies-hypotonia-seizures syndrome 3	Homozygous
RaPS_11	*WT1*	607 102	WT1-related nephropathy	De novo
RaPS_12	*GLDC*	238 300	Glycine encephalopathy	Homozygous
RaPS_15	*RRM2B*	604 712	Mitochondrial DNA depletion syndrome	Compound heterozygote
RaPS_16	*NSD1*	606 681	Sotos syndrome	De novo
RaPS_21	*TBCE*	604 934	Hypoparathyroidism-retardation-dysmorphism syndrome	Homozygous
RaPS_24	*CC2D2A*	612 013	Joubert syndrome 9	Compound heterozygote
Secondary findings				
RaPS_18	*BCHE*	177 400	Butyrylcholinesterase deficiency	Homozygous
Diagnosis made outside of RaPS				
RaPS_04	*EIF4A3*	268 850	Richieri-Costa-Pereira syndrome	Homozygous
RaPS_08	*IL2RG*	308 380	Severe combined immunodeficiency, X-Linked	X-Linked Recessive

Ten diagnoses were made as a result of WGS through RaPS, all of which explain the primary clinical findings. In one case (RaPS_18), a secondary finding of homozygous *BCHE* mutations was identified and fed back to the referring team as it was deemed clinically relevant. Two molecular diagnoses were found outside of RaPS; a patient with a known mutation in *IL2RG* (RaPS_08) was recruited to RaPS to investigate dual pathology. The *IL2RG* mutation was confirmed, but no second molecular diagnosis was made. In RaPS_04, a homozygous 5′UTR expansion not detected by WGS was identified in *EIF4A3* by a different group.

In addition to the diagnostic variants identified in phase I, a diagnostic 5′UTR expansion in *EIF4A3* was identified in an individual (RaPS_04) by a collaborative group^27^ and not detected by our WGS analysis. In a further case, we confirmed a previously identified *IL2RG* variant in a proband with immune deficiency. The referring team had requested RaPS analysis with a suspicion that there may be a second cause of the observed clinical features; however, no additional putatively causal variants were identified (table 1).

In phase II analysis, a secondary finding was identified in one individual (RaPS_18). This comprised a homozygous variant in *BCHE.* Variants in *BCHE* are associated with postanaesthetic apnoea (table 1).

#### Timelines for diagnosis

**Figure 3:**
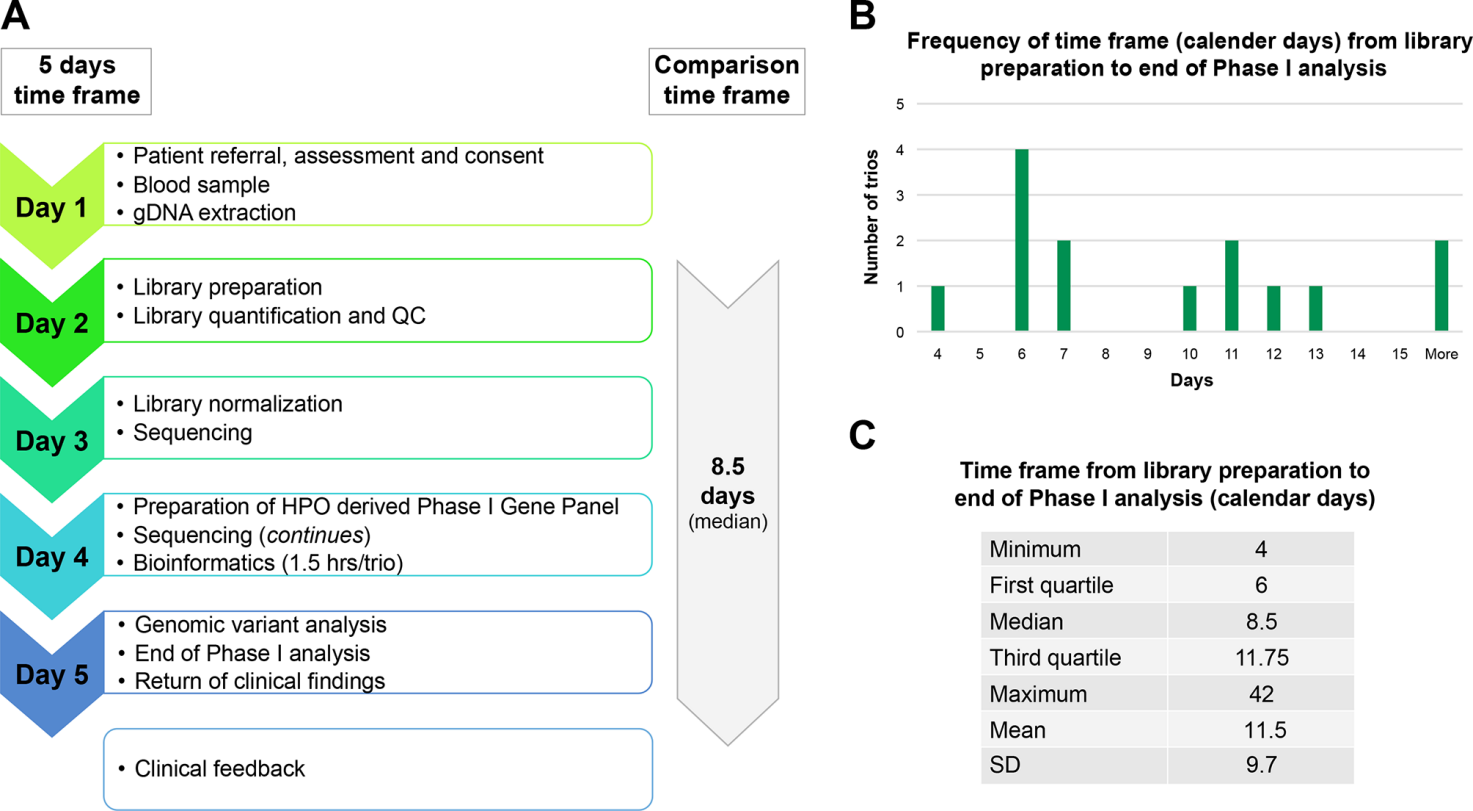
RaPS Time Frame for the last 14 cases. (Note, the first 10 cases were used for proof of principle and establishment of the workflow). (A) The 5 calendar days time frame was achieved as indicated on the left panel. *To provide a comparison with the time frames published by previous studies,^13–16^ we have calculated the median time frame of the last 14 cases from the time library preparation was initialised. Note that the timeframe to ascertain patients was variable and depended on a number of factors such as availability of parents for consenting. (B) Histogram of time frame of genomic sequencing calculated from library preparation to return of clinical findings. Weekends, holidays and delays due to reagents failure or unavailability of sequencers are not excluded from the time frame to reflect real-life working conditions. (C) Table shows the quartile distribution of time frame (calculated from library preparation to return of clinical findings).

The shortest time taken to complete the full workflow (from consent to return of provisional diagnosis) was five calendar days (RaPS_11). To allow a comparison with previous studies,^13–15^ we also measured the time to diagnosis for the last 14 trios from the point library preparation began to return of the provisional result. The shortest time for this time period was four calendar days with a median time of eight calendar days (figure 3). This timeline reflects ‘real life’ and is based on the standard working hours of a diagnostic laboratory and includes technical delays caused by reagent failure or lack of availability of sequencers. The turnaround time for the first 10 ‘proof of principle’ cases were much more variable while systems and workflow was being established. Other factors that resulted in an increased time to diagnosis include delays before the library preparation started and included the non-availability of a parent for consent or blood draw.

#### Impact of results on clinical management

In all families where a genetic diagnosis was achieved, diagnosis enabled counselling about prognosis, avoidance of unnecessary investigations and informed recurrence risk. In three individuals (RaPS_02, 11 and 16), a rapid diagnostic result had an immediate impact on the individual’s clinical management.

In the first individual, a molecular diagnosis of *COL3A1* (RaPS_02) associated with vascular Ehlers–Danlos syndromes (EDS) helped explain the presence of a ruptured spleen in this individual; prior to this genetic diagnosis, child protection concerns had been raised. The second individual, RaPS_11, who presented with renal failure, was found to have a de novo *WT1* mutation. This genetic diagnosis explained the renal phenotype and also informed the need for bilateral nephrectomy to prevent the development of Wilms tumours that are frequently associated with *WT1* mutations.^28^ Finally, the broad approach was especially successful in diagnosing RaPS_16 with Sotos syndrome, an overgrowth disorder (MIM117550). This individual was severely ill with hyperinsulinaemia and multisystem involvement. The diagnosis of Sotos syndrome was unlikely to have been made for many months in this individual as the clinical features were atypical. A diagnosis of Sotos syndrome assisted in the endocrine management of the hyperinsulinaemia by making further planned investigations unnecessary and advising that this was likely to be self-limiting.

## Discussion

We have developed a robust and readily adoptable protocol for achieving rapid end-to-end WGS-based analysis to support the diagnosis of critically ill children. Our workflow comprises detailed inclusion criteria, clinical data capture using HPO terms, rapid DNA extraction and WGS, a rapid bioinformatics analysis pipeline and tiered variant reporting.

We have successfully applied this workflow in critically ill children on an intensive care unit in a UK NHS setting and obtained a diagnostic rate of 42% (table 1), with the shortest time taken to reach a provisional diagnosis being just 4 days (figure 3). In three individuals (RaPS_02, 11 and 16), the identification of a diagnostic variant changed the immediate clinical management. In all cases, a diagnosis enabled accurate genetic counselling and disease-based management in all families.

Rapid feedback required a close working relationship between the multidisciplinary teams and for all laboratory and computational systems to be coordinated. A critical part of our workflow was the implementation of phased variant analysis and reporting to facilitate identification of likely causal variants. For successful phased variant reporting in our study, comprehensive phenotypic data captured as HPO terms was required. This enabled rapid generation of appropriate gene panels to clinically assess pathogenicity of variants identified. An example of the clinical utility of a rapid gene panel is highlighted by our analysis of individual RaPS_11, in whom we identified a *WT1* mutation. Typically, specific *WT1* testing would take on average 8 weeks. However, in this individual, the genetic differential diagnosis was wide and in the absence of RaPS WGS, routine genetic testing would have been initiated and likely included a WES gene panel with an expected turnaround time of 4 months. Furthermore, we demonstrate the utility of WGS over WES by the diagnosis made in individual RaPS_24 in whom we identified a compound heterozygous mutation in the gene *CC2D2A* comprising a coding variant and a multi exon-spanning structural variant (inversion) that would not have been identified with WES (online supplementary figure 5).

All diagnostic variants, including the previously confirmed *IL2RG* variant in RaPS_08, were identified in phase I of our tiered reporting system demonstrating its diagnostic utility (figure 1). We additionally identified a secondary finding of a homozygous *BCHE* variant in phase II analysis. In this individual, a cholinesterase assay confirmed the functional impact of the variant. Although this finding was not relevant to the underlying complex phenotype of this individual, it was assessed as important to report back to the clinical team as a secondary finding. This individual underwent several surgical procedures and therefore knowledge that post-anaesthetic apnoea was a risk with certain anaesthetic agents changed their clinical management, thus significantly reducing the risk of postanaesthetic apnoea.

A pivotal part of our data analysis pipeline that enabled rapid diagnosis was the use of fast data processing software for mapping and variant calling (Genalice, online supplementary material 3). Using this system, we were able to significantly reduce the processing time from raw sequence data (FASTQ format) to text files containing lists of variants from the reference sequence (variant call format files)^29^ from up to 144 hours (using a standard GATK pipeline) to 60 min per trio. To ensure the increased processing speed did not adversely affect the accuracy of variant calls, we processed the Genome in a Bottle reference sample under exactly the same conditions as our RaPS samples (online supplementary material 3).^30^ Furthermore, the use of Ingenuity Variant Analysis software^31^ for the annotation and filtering of variants decreased the time taken for interpretation by allowing us to apply our phased variant analysis models to the data (figure 1).

It is important to distinguish the difference between our study and that of other groups who have also performed rapid WGS on critically ill children.^14–17^ In the studies by the Kingsmore group, the time to diagnosis was far quicker owing in part to the manufacturer reconfiguration of the sequencer used and their protocol requiring staff to be available to perform each stage on a non-stop 24-hour cycle. While in the study by van Diemen and colleagues, the analysis of variant data was greatly simplified by analysing a predetermined list of 3426 genes in all samples. It is also unclear how best to compare our time scales to previous studies as in the calculation of total time, they assume no time interval between the various steps of the protocol. Here, we describe a protocol using off-the-shelf reagents and equipment, which fits into the standard working practices of a diagnostic laboratory. We also combine the benefits of a tiered analysis strategy based on a bespoke WGS panel while also affording the option of broader unbiased analysis if a diagnosis is not forthcoming.

At present, we calculate the cost of reagents and software required to run a rapid WGS trio ranges from £6105 (2× Nextseq) to £8605 (1× Nextseq, 1× Hiseq) but this is likely to fall and needs to be considered in the context of the cost of an ICU bed, estimated to be £4500/day. This cost also compares favourably with the most recent study by Farnaes and colleagues,^16^ where the full economic cost of running a trio was estimated to be $17 579 (~£13 000) and resulted in a net cost saving from 42 families of $128 554. In the UK, there are no diagnostic WGS tests yet available through the UK Genetic Testing Network that can be used to compare our costs; nevertheless, for reference, a whole exome analysis for a single family currently costs £1500 and takes 112 calendar days to return findings. A full health economics study would be beneficial to extrapolate the UK specific health benefits of a rapid diagnosis (vs one taking several months) in this group of severely ill complex individuals as a number of studies have shown sequencing to be cost-effective in other healthcare systems.^10 16 32^

In the future, all ill patients with suspected genetic disorders will likely have access to WGS. In the UK, the 100KGP is developing the infrastructure required to deliver WGS on a population scale.^13^ Studies such as ours are therefore vital in: demonstrating the utility of rapid WGS for particular patient groups, overcoming the challenges of integrating the academic, diagnostic and clinical teams and finally to produce a workflow compatible with the strict regulations required for delivering an accredited genetic diagnosis. For the present study, we are grateful for the funding we receive in the form of a National Institute of Health Research Biomedical Research Centre grant held jointly between Great Ormond Street Institute of Child Health (GOSICH) and Great Ormond Street Hospital (GOSH) as this has allowed us to develop this workflow in collaboration with our clinical and diagnostic colleagues. Nevertheless, a major challenge we have endured in this project is that it required the use of sequencing machines purchased primarily to perform routine diagnostic testing that restricted our ability to scale up the number of patients we can process to no more than one a week.

In the future, rapid WGS for critically ill children will almost certainly become a routine test for the NHS but until then it is important to select carefully those who will benefit most. Given the costs involved in managing critically ill individuals, a rapid genetic diagnosis in this group may ultimately be the most cost-effective option for the NHS and other healthcare providers.

In summary, we have presented a sustainable end-to-end workflow for using WGS to rapidly diagnose critically ill individuals with likely monogenic genetic disorders. Such a workflow uses off-the-shelf products and could readily be adopted by other diagnostic centres.
