# Supplementary material for: Rapid Paediatric Sequencing (RaPS): comprehensive real-life workflow for rapid diagnosis of critically ill children
Source: J Med Genet. 2018 Jul 26;55(11):721–8. doi: 10.1136/jmedgenet-2018-105396 (PMC6252361; doi:10.1136/jmedgenet-2018-105396)
Supplement: Supplementary data [file jmedgenet-2018-105396supp001.pdf]

Rapid Paediatric Sequencing (RaPS): comprehensive real-life workflow for rapid diagnosis of critically ill children

Lamia Mestek-Boukhibar[1], Emma Clement[2], Wendy Jones[2], Suzanne Drury[3], Louise Ocaka[1], Andrey Gagunashvili[1], Polona Le Quesne Stabej[1], Chiara Bacchelli[1], Nital Jani[1], Shamima Rahman[4], Lucy Jenkins[5], Jane Hurst[2], Maria Bitner-Glindzicz[4], Mark Peters[6], Philip L Beales[1] & Hywel J Williams[1]

Supplementary Information

[1]*GOSgene, Genetics and Genomic Medicine, UCL Great Ormond Street Institute of Child Health, London, United Kingdom*
[2]*Clinical Genetics and Genomic Medicine, Great Ormond Street Hospital for Children NHS Foundation Trust, London, United Kingdom*
[3]*Congenica Ltd, Bioinnovation Data Centre, Wellcome Genome Campus, Cambridge, United Kingdom*
[4]*Genetics and Genomic Medicine, UCL Great Ormond Street Institute of Child Health, London, United Kingdom*
[5]*NE Thames Regional Genetics Laboratory, Great Ormond Street Hospital, London, United Kingdom*
[6]*UCL Great Ormond Street Institute of Child Health and Great Ormond Street NHS Foundation Trust, London, United Kingdom*

Table of Contents

Supplementary text S1 Whole genome sequencing 3
Supplementary text S2 Variant analysis 4
Supplementary text S3 Sequencing analysis 6
References 7
Figure S1 Specialties involved in care of probands recruited to RaPS 8
Figure S2 Age of probands recruited to RaPS at the time of whole genome sequencing 9
Figure S3 RaPS referrals 10
Figure S4 Variant breakdown overview 11
Figure S5 Identification and validation of *CC2D2A* inversion 12
Table S1 Patients' phenotypes at the time of referral 13
Table S2 Number of variants per trio 16
Table S3 Sample mean coverage and percentage of whole genome covered at the indicated read depth in both parents and proband 18
Table S4 Gene variants shortlisted in each patient 20

Supplementary Text S1.

Whole genome sequencing

Genomic DNA (gDNA) preparation: Whole genome sequencing was performed on a trio (father, mother and proband). gDNA was extracted from blood sample using Chemagic-STAR (Hamilton, USA) in a diagnostic accredited lab (NE Thames Regional Genetics Lab). High quality gDNA was used for whole genome library preparation. One μl of gDNA was run on 1% agarose gel to confirm absence of degradation. gDNA concentration was measured using Qubit dsDNA Broad Range Assay Kit (Thermo Fisher Scientific, Waltham, MA). DNA was diluted to 1.1 μg in total volume of 55 μL in HT1 buffer (Illumina, USA) and transferred to Covaris 50 μL individual tubes (Woburn, MA). gDNA was sheared to 350 bp using E220 Focused-ultrasonicator (Woburn, MA) for 60 seconds with the following parameters: target peak BP 400, peak incident power 140, duty factor 10% and 22 cycles of burst. Successful shearing was assessed on 1% agarose gel prior to starting library preparation.

Library preparation: Whole genome gDNA libraries were prepared using TruSeq DNA PCR-Free Library Prep (Illumina, USA) following manufacturer advice starting with 1 μg of sheared gDNA (in 50 μL). Libraries were single indexed using Illumina's indexed adapters (Set A FC-121-3001 or Set B FC-121-3002, Illumina, USA). Library concentration was measured using by quantitative Polymerase Chain Reaction (qPCR) (KAPA Biosystems, Roche, Basel, Switzerland) following manufacturer's advice. Briefly, 2 μL of library was diluted 10 000$\times$ and 20 000$\times$ in dilution buffer (100 μL Tween 20, 2 mL 1 M Tris and 198 mL dH_2O) and incubated overnight. qPCR was performed in triplicates in a total volume of 16 μL each and run on Applied Biosystems 7300 qPCR machine (Thermo Fisher Scientific, Waltham, MA).

Library normalization and sequencing: Libraries were normalised to 2 nM with Tris $\cdot$ HCl (10 mM pH 8.5) supplemented with 0.1% Tween 20. Libraries were denatured with 0.2 N NaOH and stabilised with 200 mM Tris $\cdot$ HCl. gDNA libraries from parents were pooled and 2.7 pM was sequenced on Illumina NextSeq 550 System with 2$\times$150 bp for 29 hours. gDNA library from proband was sequenced on double flow cell on Illumina HiSeq 2500 System (Rapid Run Mode) with 9 pM loading concentration and 2$\times$150 bp for 30 hours. For the last two trios (RaPS_23 and PaPS_24), proband samples were sequenced on NextSeq 550 System.

Supplementary Text S2.

Variant analysis

Variant analysis and filtering was performed using Ingenuity Variant Analysis (IVA) software (QIAGEN; *https://variants.ingenuity.com/*).

Summary of variant filtering and shortlisting: VCF files for each trio were uploaded to IVA and sample relationships were annotated using a ped file (father, mother and proband) where unaffected parents were used as controls and proband as a case.

The number of variants as shown in Supplementary Figure S4 is the total number of variants per trio to which pre-filters were then applied: first, common variants were filtered out by excluding those that are present at minor allele frequency (MAF) >0.5% in the public databases as unlikely to be causative; second, a filter was applied to only keep the variants that were predicted to effect protein function (missense variants, stop gains or losses, frameshifts, small insertions and deletions (indels) as well as variants affecting splicing sites present at ± 7 nucleotides from exon-intron junctions).

Variant short listing was performed in Phases (I, II and III) as described in the main text Methods section. In Phase I analysis, variants that fell within genes that are part of Phase I gene list were kept the rest were excluded. These variants were then sorted based on mode of inheritance by applying a genetic filter in IVA to keep only variants that are present in the proband. The same principle was applied to Phase II and Phase III analysis. By applying this cascade of filters, large amount of variants were excluded to only focus on those that are relevant to patient and clinical manifestation.

Integrated Genome Viewer (IGV) was used in parallel to supplement the variant shortlisting. For each shortlisted variant (an average of 7–10 per trio) the genomic location was viewed in IGV to determine the quality of the read.

1. Pre-filtering step:

a. Common variants with MAF >0.5% in 1000G (1000 Genomes Project Consortium et al., 2015), ExAC (Lek et al., 2016) and Exome Variant Server (*evs.gs.washington.edu/EVS/*) databases were filtered out. For homozygous and hemizygous variants in proband, MAF is increased to $\leq$10% and variants with no homozygotes/hemizygotes in ExAC were investigated.

b. Effect of variant on protein function was set to include predicted pathogenic, likely pathogenic and uncertain significance (benign or likely benign variants are investigated if further evidence of pathogenicity was available).

c. Variants associated with loss of function were kept: frameshift causing, in-frame insertions/deletions, missense and splice site (± 7 nucleotides).

d. 5′ UTR and 3′ UTR variants were also investigated for genes known to be disease-causing or with compelling evidence for candidate genes.

2. Phase I analysis: This comprised setting a gene panel as a filter to investigate genes associated with the patients reported phenotypes as the first line of investigation. The gene panel was constructed by converting clinical phenotypes to HPO terms retrieving associated genes from different sources: The Genomics England PanelApp (*https://panelapp.*

genomicsengland.co.uk/), Phenotips (https://phenotips.org/), OMIM Gene Map (https://omim.org/search/advanced/geneMap), established panels from NE Thames Regional Genetics Lab, Great Ormond street Institute of Child Health-UCL experts and literature search in PubMed.

3. **Phase II analysis workflow:** This consisted of variants in disease-associated genes from OMIM (https://omim.org/search/advanced/geneMap) and DDG2P (Firth et al., 2009) databases.

4. **Phase III analysis workflow:** This is performed when Phase I and Phase II variants are exhausted and or when a variant is interesting from a research point of view and consists of variants that pass the following set of criteria: a. Effect on protein function is likely damaging b. Supportive research based evidence from the literature with link to patient phenotypes

Mode of inheritance: Here the "mode of inheritance" (MOI) term is used to refer to the inheritance of the variant in the proband and not a disease. MOI are prioritized based on parents consanguinity and family history to cover: autosomal recessive (homozygous in proband and heterozygous in parents), compound heterozygous, *de novo*, X-linked dominant (XLD) and X-linked recessive (XLR) to include hemizygous in male patients. The rapid mode of sequencing often does not yield read depth that permits detection of mosaicism, however, if suspected then Sanger sequencing is performed. Specifically, in the scenario where we had a recessive gene in which only one potentially pathogenic SNV variant was identified from the VCF file, the entire gene was inspected using IGV to search for a potential second variant that was not called using our standard analysis pipeline (such as possible deletions, translocations, or inversions). Detecting structural variants on IGV requires a trained professional, and a full IGV manual is freely accessible: http://software.broadinstitute.org/software/igv/UserGuide. Briefly, we searched for an obvious drop in coverage as an indication of deletion, and we inspected the orientation of the paired-reads which are colour coded (whole genome sequencing was perfumed as paired-end sequencing as indicated in our methods section), and so reads face each other. Paired-reads that have same orientation appear in different colour and are an indication of inversion which can be verified by Sanger sequencing.

Supplementary Text S3.

Sequencing analysis

Read mapping and variant calling: Basecalling of raw sequencing reads was performed on BaseSpace Sequence hub (*http://basespace.illumina.com*). Fastq files for each individual were downloaded from BaseSpace, and reads from different lanes were merged together. Mapping and variant calling were performed using a Genalice appliance running Genalice Map 2.5.5 including Mapping, Variant Calling and the Population Calling modules for trio analysis (Genalice Core BV, Netherlands). Human genome build 37 (GRCh37, hg19) and Genalice default configuration files were used for WGS mapping, and trio variant detection. Aligned reads were stored in the GAR format (Genalice Aligned Reads), using less than 5 GB per sample. Variants were stored in a GVM (Genalice Variant Map) per trio, using less than 200 MB per sample. A standard multi sample VCF with Mendelian inheritance annotation using Context Based Call Enhancement was extracted from each GVM.

Processing speed comparison: To compare the processing speed between using a Genalice appliance and open source software we calculated the time taken to analyse a randomly chosen trio from our study. The Genalice analysis is as described above and for the comparison we used BWA-MEM (Li and Durbin, 2010) for read mapping and GATK (DePristo et al., 2011; McKenna et al., 2010) for variant calling with the analysis run on our in-house UCL high performance computing cluster.

Variant calling accuracy comparison: To explore whether the quality of the variants called by Genalice and open source software were comparable we utilised the reference DNA sample NA12878 (*http://jimb.stanford.edu/giab/*). We prepared this sample for sequencing using the exact same protocol as that used for all our RaPS trios and sequenced it under the same conditions as we did for proband samples. The fastq files were then processed under the conditions detailed above and the resultant VCF file was processed to calculate the SNP-precision, SNP-recall and SNP-Fscore (Zook et al., 2014). High confidence regions values obtained are indicated below:

True-positive	False-positive	False-negative	SNP-precision	SNP-recall	SNP-Fscore
3,048,709	49,430	103,717	0.984	0.9671	0.9755

References

DePristo, M. A., E. Banks, R. Poplin, K. V. Garimella, J. R. Maguire, C. Hartl, A. A. Philippakis, G. Del Angel, M. A. Rivas, M. Hanna, et al. (2011). A framework for variation discovery and genotyping using next-generation dna sequencing data. *Nature Genetics 43*(5), 491–498.

Firth, H. V., S. M. Richards, A. P. Bevan, S. Clayton, M. Corpas, D. Rajan, S. Van Vooren, Y. Moreau, R. M. Pettett, and N. P. Carter (2009). DECIPHER: database of chromosomal imbalance and phenotype in humans using Ensembl resources. *The American Journal of Human Genetics 84*(4), 524–533.

Lek, M., K. J. Karczewski, E. V. Minikel, K. E. Samocha, E. Banks, T. Fennell, A. H. O'Donnell-Luria, J. S. Ware, A. J. Hill, B. B. Cummings, et al. (2016). Analysis of protein-coding genetic variation in 60,706 humans. *Nature 536*(7616), 285–291.

Li, H. and R. Durbin (2010). Fast and accurate long-read alignment with Burrows–Wheeler transform. *Bioinformatics 26*(5), 589–595.

1000 Genomes Project Consortium et al. (2015). A global reference for human genetic variation. *Nature 526*(7571), 68–74.

McKenna, A., M. Hanna, E. Banks, A. Sivachenko, K. Cibulskis, A. Kernytsky, K. Garimella, D. Altshuler, S. Gabriel, M. Daly, et al. (2010). The genome analysis toolkit: a mapreduce framework for analyzing next-generation dna sequencing data. *Genome Research 20*, 1297–1303.

Zook, J. M., B. Chapman, J. Wang, D. Mittelman, O. Hofmann, W. Hide, and M. Salit (2014). Integrating human sequence data sets provides a resource of benchmark SNP and indel genotype calls. *Nature Biotechnology 32*(3), 246–251.

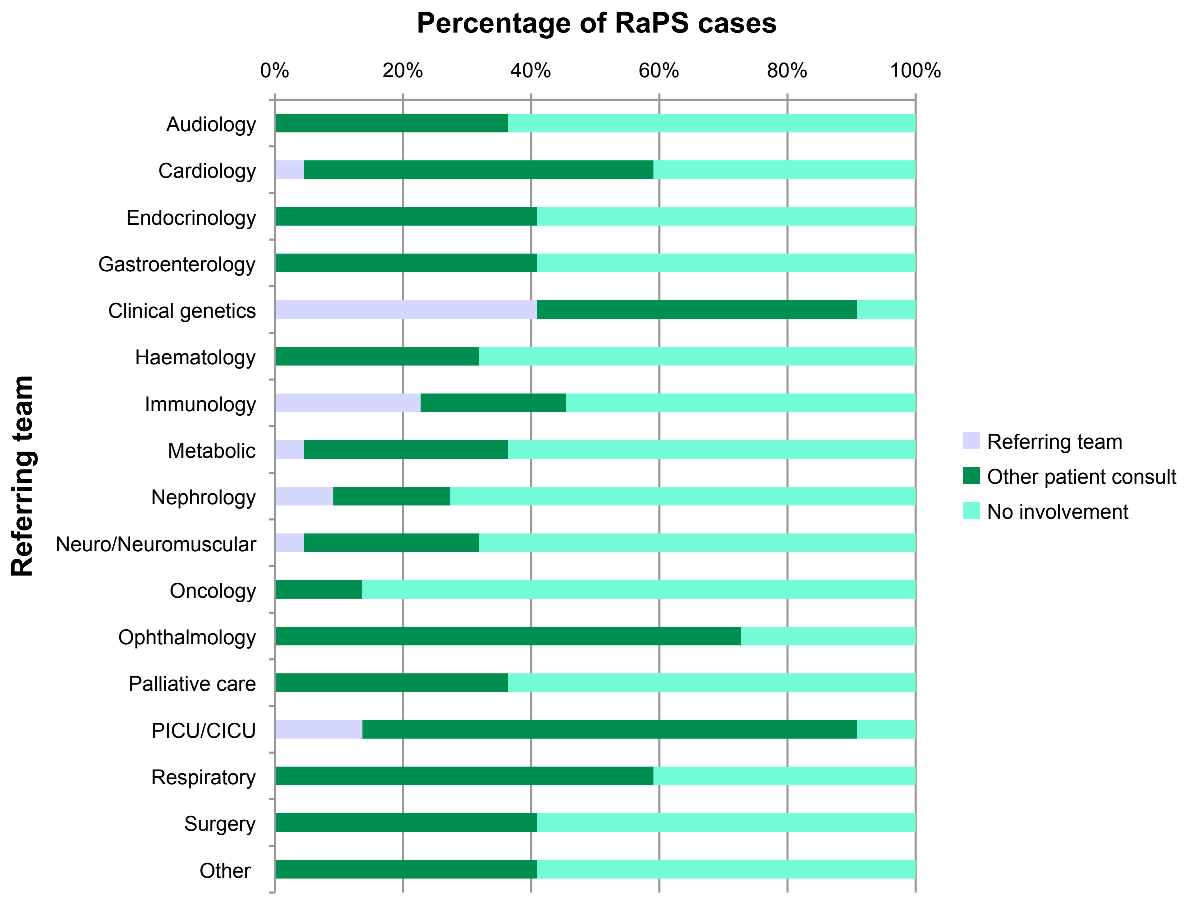

Figure S1. Specialties involved in care of probands recruited to RaPS

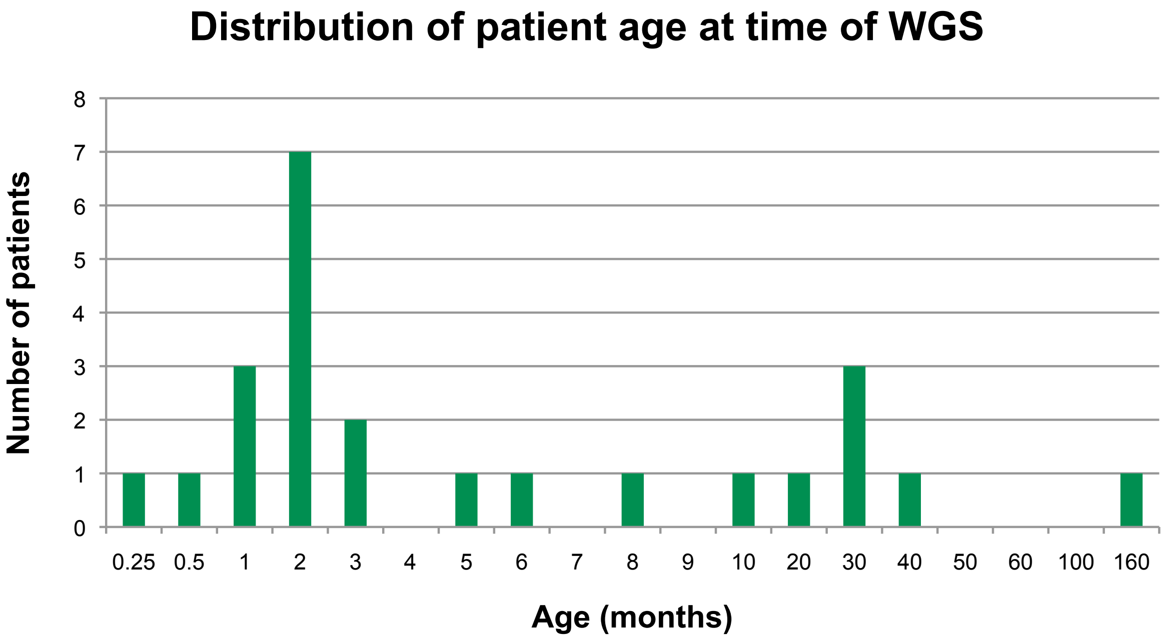

Figure S2. Age of probands recruited to RaPS at the time of whole genome sequencing

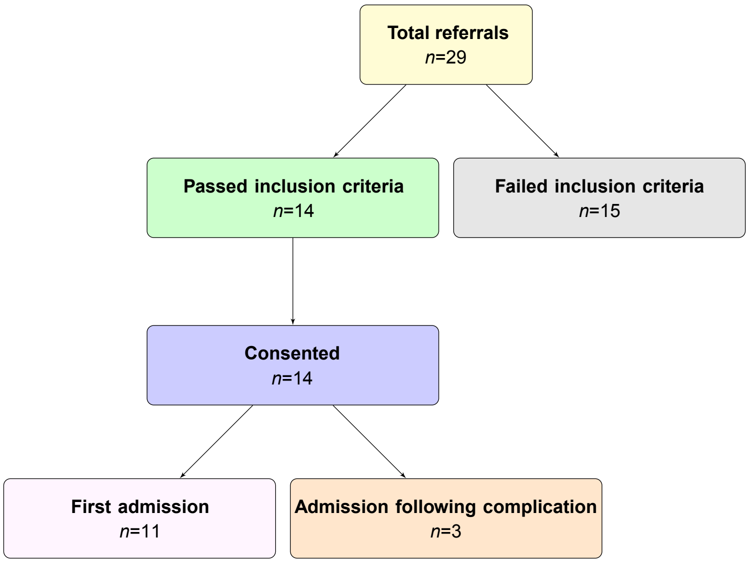

Reason for exclusion	Number of patients
Not a trio	2
High risk of imminent demise	1
Not genetic (diabetic embryopathy)	1
Unlikely to change clinical management	2
Other testing suggested	3
Extensive testing already performed	1
Technical reasons	5

Figure S3. RaPS referrals. The figure depicts the number of individuals referred to RaPS and whether they have passed the inclusion criteria or not. The accompanying table lists the reasons for exclusion for individuals who were not included in RaPS.

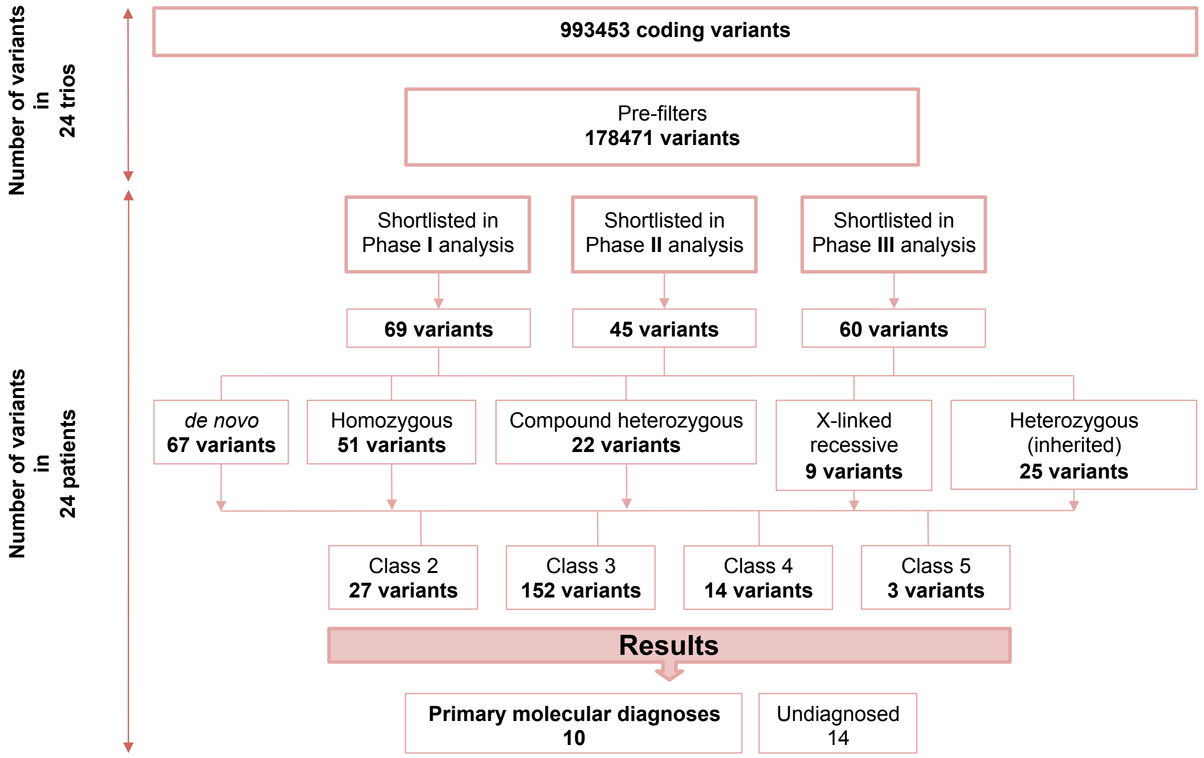

Figure S4. Variant breakdown overview. Schematic breakdown of number of variants in 24 trios in different steps of the RaPS workflow. In Phase I, the number of genes filtered is different in each trio. Phase II is composed of genes from GGD2P (1 643genes) and OMIM Morbid Genes (7 737 genes) databases and is standard across all trios. The RaPS variant analysis workflow is phased to prioritise likely causative gene variants. Note that compound heterozygous and homozygous variants are counted once. Refer to Supplementary text S2 for detailed description of variant filtering and shortlisting.

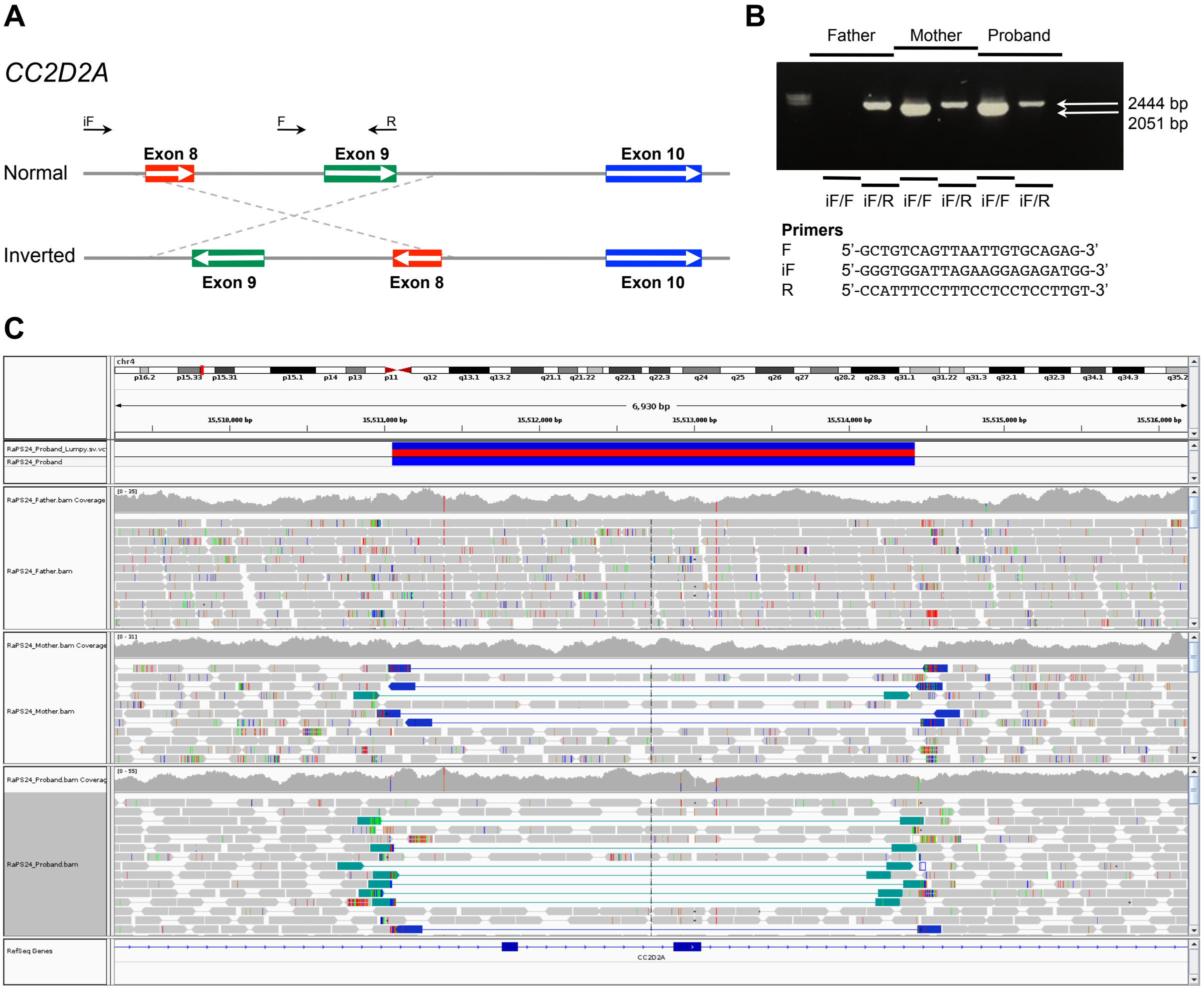

Figure S5. Identification and validation of *CC2D2A* inversion. **A** Schematic diagram depicting the reference configuration of part of *CC2D2A* gene (only exons 8–10 are shown for simplicity) and an inversion spanning exons 8 and 9. **B** Mother and proband are heterozygous carriers of the inversion and both primer pairs iF/F and iF/R generate a PCR product of 2 051 bp and 2 444 bp, respectively. Father is not a carrier and only primer pair iF/R generates a PCR product of 2 444 bp. Sequencing of the iF/F PCR product in both mother and proband identified the exact location of break points (Chr4:15510661–15514794; hg19). **C** IGV screenshot showing the discordant reads (coloured sea green and blue) in both mother and proband.

Table S1. Patients' phenotypes at the time of referral

Patient	Age at time of whole genome sequencing (sex)	Phenotypes
Patient 1	1 year 7 months 15 days (F)	Absent B cells, multiple septic episodes, disseminated infection with multiorgan involvement (lungs, brain, liver, gut), granulomatous lower back collection, tubular leak with occasional hypernatremia, hypertension, bicytopenia (low platelets and Hb), generalised oedema, capillary leak, fragile skin
Patient 2	12 years 5 months 1 days (F)	Easy bruising, failure to thrive, splenic rupture, blue sclerae, joint hypermobility
Patient 3	0 years 5 months 18 days (F)	Talipes, fasciculation, abnormal dopamine turnover, abnormality of the brain, small cerebellum, simplified gyral pattern, contractures, low set ears, alveolar cleft gums, stiffness, small nasal cleft, polyhydramnios
Patient 4	0 years 7 months 28 days (M)	Craniofacial abnormalities, abnormality of the limbs, talipes, micrognathia, camptodactyly, short halluces
Patient 5	0 years 1 month 30 days (M)	Severe combined immunodeficiency (thymic), cerebellar dysplasia, delayed cortical development, visual impairment, cleft lip/palate, craniosynostosis, hypoparathyroidism
Patient 6	0 years 2 months 18 days (F)	High platelet count, hypoplastic aortic arch, double outlet right ventricle, bicuspid pulmonary valve, ventricular septal defects, severe combined immunodeficiency (thymic), cerebellar dysplasia, delayed cortical development, visual impairment, cleft lip/palate, craniosynostosis, hypoparathyrodism, radial aplasia, absent spleen, micrognathia, coarctation of the aorta, tracheobronchomalacia, gastro-oesophageal reflux disease, doubly committed ventricular septal defect (VSD) with additional small apical VSDs, left anterior descending artery from right coronary artery, periauricular pits, long fingers and toes, triphalangeal thumbs, hypermetropic astigmatism, intermittant exotropia
Patient 7	0 years 6 months 9 days (F)	Global developmental delay, seizures, cerebral haemorrhage, visual impairment, bilateral hydronephrosis, small patent foramen ovale, anaemia, recurrent upper tract infections, fungal upper tract infection, ectopic ureters, abnormal lymphocyte count (low) but normal proportion of T-cells, B-cells and NK-cells
Patient 8	2 years 7 months 16 days (M)	Multiple serositis, severe hypotonia (trunk and lower limbs), mild calcineurin inhibitor related microangiopathy, fragmentocytes, thrombocytopaenia, graft versus host disease of the gut, chronic inflammatory, gut epithelial damage, microangiopathy, multiple sclerosis, severe combined immunodeficiency, immune cell abnormalities, neutropenia, viral infection, fluid retention, capillary leak, blood pressure, hypoalbuminaemia
Patient 9	1 year 9 months 14 days (F)	Pulmonary hypertension, alveolar hypoplasia, upper respiratory tract infection, anaemia, renal failure, thrombyctopaenia, status epilepticus, encephalitis, metabolic acidosis respiratory failure, impaired cardiac function, atypical hemolytic-uremic syndrome

Continued on next page

Table S1. *(continued from previous page)*

Patient	Age at time of whole genome sequencing (sex)	Phenotypes
Patient 10	5 years 1 month 13 days (M)	Recurrent upper respiratory tract infections, tonsillitis, hypertriglyceridemia, hematochezia, splenomegaly, thrombocytopenia, neutropenia, anaemia due to reduced life span of red cells, colitis, increased serun ferritin, fever, lymphadenopathy, retinal vein occlusion, Epstein-Barr virus-induced hemophagocytic lymphohistiocytosis
Patient 11	0 years 1 month 2 days (M)	Acute oligonuric kidney injury, ossification defect of the skull
Patient 12	0 years 0 months 16 days (M)	Nonketotic hyperglycinaemia, continuing severe encephalopathy
Patient 13	0 years 10 months 16 days (F)	Primary immune deficiency, atypical haemolytic uremic syndrome
Patient 14	2 years 4 months 11 days (F)	Intrauterine growth retardation, failure to thrive, skeletal dysplasia, lung dysplasia, mild hypotonia resulting in delayed swallow and nocturnal hypoventilation, small ventricula septal defect, small subaortic shelf, suspected primary immunodeficiency, Epstein-Barr virus-driven high grade lymphoma, iliac crest serration, lacy pelvis, coarse facial features, bulbar palsy, platyspondyly, coronal notching at the lumbar region, thoraco lumbar kyphosis
Patient 15	0 years 3 months 7 days (M)	Profound bilateral sensorineural hearing loss, hypotonia, failure to thrive despite good feeding, metabolic acidosis, lactic acidosis
Patient 16	0 years 2 months 1 day (M)	Cardiac abnormalities, small patent ductus arteriosus, neurological problems, right duplex kidney, intracerebral cyst, soft dysmorphic features, adducted thumbs, slanting eyes, right ventricular hypertrophy, hyperinsulinemic hypoglycemia, multiple thrombi including large inferior vena cava clot and cerebral infraction, cystic encephalomalacia, deranged liver function, ontractures
Patient 17	0 years 2 months 24 days (M)	Antenatal hydrops fetalis, pancytopenia, hepatosplenomegaly, thrombocytopenia, very high ferritin, skin rash, XIAP deficiency, neonatal hypothyroidism, hypochloraemic metabolic acidosis, histiocytosis on bone marrow biopsy, increased circulating low-density lipoprotein levels, high triglycerides, low HDL cholesterol levels, abnormal liver function tests, congenital dislocation of hip, inguinal hernia, rhizomelic short limbs, blue sclerae, short stature

Continued on next page

Table S1. *(continued from previous page)*

Patient	Age at time of whole genome sequencing (sex)	Phenotypes
Patient 18	0 years 0 months 27 days (M)	Bilateral cleft lip/palate, horseshoe kidney, ambiguous genitalia, hypospadias, undescended testes, hypoplastic aortic arch, patent ductus arteriosus, atrial septal defect, abnormal myocardium, coloboma (optic disk and iris), dilation of intrahepatic and extrahepatic bile ducts, right pleural effusion, microcephaly, hypertelorism, broad nasal bridge, exorbitism, arched eyebrows, low set ears, hypertrichosis, short neck with redundant skin at the nape, rhizomelic shortening of the upper limbs, clinodacyly, syndactyly, thickened skin over fingers, prominent anterior plantar pad, overlapping toes, short penis, scrotal oedema, failure to thrive, immune deficiency, coarctation of aorta, cystic, renal dysplasia, hearing impairment, vertebral abnormalities (butterfly vertebrae at T2), choledochal cyst in liver, deranged thyroid functional tests (lowT3)
Patient 19	0 years 2 months 5 days (F)	Heart defects, atrial septal defect, patent ductus arteriosus, mild aortic stenosis, dysplastic multicystic kidneys, hypertrichosis, natal tooth, small jaw, inverted nipples, restriction of elbow extension, conjugated bilirubinaemia
Patient 20	0 years 8 months 26 days (M)	Congenital heart defects (ventricular septal defect and double outlet right ventricle), hypoplastic corpus callosum, small pons, small cerebellum, severe tracheobronchomalacia, diaphragmatic eventration, heart block, inguinal hernia, hyperinsulinism, abnormal fat distribution
Patient 21	0 years 1 months 25 days (M)	Ambiguous genitalia, bifid scrotum hypocalcaemia, hyponatremia, hypoglycaemia, patent ductus arteriosus, hyperbilirubinemia, microcephaly, thin ribs, short limbs, brachydactyly, rocker, bottom feet, recurrent infection, short toes, small phallus, depressed nasal bridge
Patient 22	0 years 3 months 3 days (M)	Congenital cataract, small pupils, dilated cardiomyopathy, lactic acidosis
Patient 23	0 years 1 month 6 days (M)	Intrauterine growth retardation, neurogenic arthrogryposis, hypotonia, scoliosis, anterior horn cell pathology
Patient 24	0 years 0 months 17 days (F)	Prenatal hydrocephalus, dystroglycanopathy, post axial polydactyly of hands, abnormal cerebellar vermis, respiratory distress

Human Phenotype Ontology terms were derived for each patient using the phenotype information shown here and used to construct Phase I gene panels.

Table S2. Number of variants per trio (frameshifts, in-frame indels, start/stop codon changes, missense variants)

RaPS trio ID	Whole genome	Coding regions					
	Number of variants per trio	Number of variants per trio	Number of variants passed pre-filter per trio	Passed Phase I per trio (number of genes in Phase I)	Shortlisted from Phase I per patient[a]	Shortlisted from Phase II per patient[a] (DDG2P, OMIM)[b]	Shortlisted from Phase III per patient[a]
RaPS_01	6,047,454	42,573	7,150	1,149 (2,642)	1	no Phase II performed[c]	no Phase III performed[c]
RaPS_02	5,489,792	40,354	6,293	325 (832)	1	no Phase II performed[c]	no Phase III performed[c]
RaPS_03	6,209,676	45,000	9,241	554 (904)	0	0	1
RaPS_04	6,962,558	48,977	15,107	913 (917)	0[d]	0	0
RaPS_05	5,954,527	41,700	6,822	97 (260)	1	no Phase II performed[c]	no Phase III performed[c]
RaPS_06	6,582,174	40,678	6,166	536 (990)	3	1	5
RaPS_07	5,810,316	41,512	6,936	231 (672)	1	2	6
RaPS_08	6,137,998	43,474	7,168	263 (756)	2	0	4
RaPS_09	6,071,727	43,206	7,295	398 (848)	3	1	1
RaPS_10	5,841,339	41,891	7,213	264 (756)	3	1	2
RaPS_11	5,826,869	42,147	6,809	36 (51)	1	1	3
RaPS_12	5,475,165	40,466	6,562	2 (7)	1	no Phase II performed[c]	no Phase III performed[c]
RaPS_13	6,000,484	41,574	7,020	89 (284)	2	1	14
RaPS_14	6,066,300	42,794	8,060	312 (756)	7	1	6
RaPS_15	6,051,267	42,536	6,794	73 (338)	1	7	4
RaPS_16	5,991,679	42,633	6,948	697 (1,921)	4	1	0
RaPS_17	5,033,594	34,458	4,815	381 (1,354)	3	3	0
RaPS_18	5,061,461	33,915	5,183	145 (424)	7	3	0

Continued on next page

Table S2. *(continued from previous page)*

RaPS trio ID	Whole genome	Coding regions					
	Number of variants per trio	Number of variants per trio	Number of variants passed pre-filter per trio	Passed Phase I per trio (number of genes in Phase I)	Shortlisted from Phase I per patient[a]	Shortlisted from Phase II per patient[a] (DDG2P, OMIM)[b]	Shortlisted from Phase III per patient[a]
RaPS_19	5,581,553	37,792	6,308	231 (374)	2	5	6
RaPS_20	6,008,553	43,131	7,261	311 (758)	1	3	4
RaPS_21	5,334,898	39,662	8,248	981 (1,848)	8	2	0
RaPS_22	4,658,755	31,974	4,698	150 (1,970)	8	3	1
RaPS_23	5,859,596	48,115	12,562	2752 (4,039)	6	8	3
RaPS_24	5,870,780	42,891	7,812	147 (284)	3	2	0
**Total**	139,928,515	993,453	178,471	8,120 (19,626)	69	45	60
**Average/trio**	5,830,355	41,394	7,436	369 (892)	3	2	3

Table shows the number of variants per trio analysis at different stages of the variant selection workflow. Data was filtered at MAF $\leq$10%, effect of variant: missense, start/stop codon change, frameshifts, in-frame indels and splice site loss $\pm$ 7 nucleotides. MAF was later adjusted according to zygosity, it was decreased to $\leq$0.5% in *de novo* and heterozygous variants, and kept at $\leq$10% in homozygous, compound heterozygous and X-linked recessive variants in which case only variants with no reported homozygous/hemizygous were considered. Shortlisting of variants was based on effect on protein function, zygosity and association with patient phenotypes. Phase I filter consisted of a phenotype-specific gene list and was generated through Genomics England PanelApp, Phenotips, OMIM, establised panels, NE Thames Regional Genetics Laboratory, GOSICH experts and literature search. The number of variants that passed Phase I gene filter are per trio. The parental genotypes were used to infer mode of inheritance in the patients and informed variants shortlisting. Shortlisted variants are thus per patient.

[a]Homozygous and compound heterozygous variants are counted once.

[b]Version downloads: DDG2P (2016) contained 1,643 genes, OMIM Morbid Genes (25-Feb-2017) contained 7,737 genes.

[c]Candidate gene identified in Phase I.

[d]5′ UTR expansion in *EIF4A3* Phase I candidate gene was not picked up by our WGS analysis.

Table S3. Sample mean coverage and percentage of whole genome covered at the indicated read depth in both parents and proband

Sample ID	Mean coverage	% bases above 10×	% bases above 15×	% bases above 20×	% bases above 30×
RaPS_1 Father	11.3	65.9	22.3	3.2	0.1
RaPS_1 Mother	9.7	50.9	10.5	0.9	0.1
RaPS_1 Proband	16.4	90.4	62.9	27.1	1.4
RaPS_2 Father	29.6	96.3	92.6	86.4	55.1
RaPS_2 Mother	9.3	46.4	8.5	0.7	0.1
RaPS_2 Proband	26.8	95.4	91.7	82.8	38.6
RaPS_3 Father	13.8	80.4	44.9	12.6	0.3
RaPS_3 Mother	14.3	84.4	48.9	14.1	0.3
RaPS_3 Proband	32.5	96.6	95	91.7	67.6
RaPS_4 Father	6.6	16.8	1.1	0.1	0
RaPS_4 Mother	8.1	32.7	3.7	0.3	0
RaPS_4 Proband	20.1	93.9	82.3	54.9	6
RaPS_5 Father	19.7	91	76.8	52	7.8
RaPS_5 Mother	23.2	94.2	86.8	69.8	20.3
RaPS_5 Proband	10.3	56.9	13.9	1.4	0.1
RaPS_6 Father	11.1	64.1	21.5	3.1	0.1
RaPS_6 Mother	12.7	76.7	33.9	6.8	0.1
RaPS_6 Proband	47.3	97	96.3	95.1	90.1
RaPS_7 Father	11.1	64.1	21.4	3.1	0.1
RaPS_7 Mother	11.8	70.4	26.3	4.3	0.1
RaPS_7 Proband	33.5	97.2	96.2	93.6	69.1
RaPS_8 Father	16.2	85.3	61.5	29.6	1.6
RaPS_8 Mother	19.6	91.9	78.9	51.8	6.4
RaPS_8 Proband	16.7	90	65.7	28.7	1
RaPS_9 Father	19.8	93.5	80.1	51.6	7.4
RaPS_9 Mother	13.1	76.1	37.9	9.9	0.2
RaPS_9 Proband	28.7	96.7	93.1	82.4	43.6
RaPS_10 Father	10.4	57.6	17.8	2.4	0.1
RaPS_10 Mother	9.5	49.9	11.9	1.3	0.1
RaPS_10 Proband	22.2	95.2	87	66.2	12.6
RaPS_11 Father	15.9	86.3	60.9	26.5	1.1
RaPS_11 Mother	10.3	56.6	14.9	1.9	0.1
RaPS_11 Proband	9.6	48.7	10.4	1.1	0.1
RaPS_12 Father	15.0	83.5	54.8	20.8	0.6
RaPS_12 Mother	11.8	69	27.9	5.4	0.1
RaPS_12 Proband	33.6	96.5	93.7	88.8	69.5
RaPS_13 Father	10.8	60.4	20.5	3.3	0.1
RaPS_13 Mother	21.4	93.7	83.8	61.5	12.3
RaPS_13 Proband	32.3	97.3	96.3	92.7	62.4
RaPS_14 Father	12.3	72.5	31.6	6.3	0.1
RaPS_14 Mother	14.1	83	46.7	13.3	0.3
RaPS_14 Proband	22.0	96.2	89.1	66	10.4

Continued on next page

Table S3. *(continued from previous page)*

Sample ID	Mean coverage	% bases above 10×	% bases above 15×	% bases above 20×	% bases above 30×
RaPS_15 Father	13.3	78.2	40.4	10.2	0.2
RaPS_15 Mother	12.4	74.6	32.1	6.3	0.1
RaPS_15 Proband	18.1	91.8	73.3	40	3.1
RaPS_16 Father	13.7	79.8	44.4	12.6	0.3
RaPS_16 Mother	14.9	85.6	53.9	18.4	0.5
RaPS_16 Proband	10.4	57	15.2	2	0.1
RaPS_17 Father	6.1	14.1	0.9	0.1	0
RaPS_17 Mother	5.3	8.5	0.4	0	0
RaPS_17 Proband	11.8	67.7	26.2	5.3	0.2
RaPS_18 Father	11.8	58.5	32.1	13.5	1
RaPS_18 Mother	9.0	42.6	15.5	3.7	0.1
RaPS_18 Proband	21.3	94.7	85.1	61.6	9.5
RaPS_19 Father	16.9	83.1	62.3	36.3	4.7
RaPS_19 Mother	15.4	77.2	53	28.1	3.6
RaPS_19 Proband	16.5	91.8	65.2	26.3	0.8
RaPS_20 Father	15.5	84.8	58.1	23.8	0.8
RaPS_20 Mother	13.0	76.8	38.1	9.6	0.2
RaPS_20 Proband	15.5	87.3	57.2	20.5	0.6
RaPS_21 Father	18.7	90.9	72.6	43.5	5.4
RaPS_21 Mother	8.4	34.7	5.8	0.6	0.1
RaPS_21 Proband	38.5	98.1	97.1	94.6	81.9
RaPS_22 Father	6.4	17.9	3	0.4	0.1
RaPS_22 Mother	5.7	13	1.6	0.2	0.1
RaPS_22 Proband	17.1	93	68.5	30.2	1.5
RaPS_23 Father	11.4	65.7	23.1	3.9	0.1
RaPS_23 Mother	14.0	82.2	44.5	13.4	0.3
RaPS_23 Proband	29.1	97.3	93.6	84.7	48.1
RaPS_24 Father	16.9	89.3	64.5	32.4	2.06
RaPS_24 Mother	14.2	82.2	46.2	15.7	0.5
RaPS_24 Proband	32.2	98.1	97.2	93.9	63.3
Parents average	13.3	67.1	38.1	17.4	2.9
Proband average	23.2	88.2	72.1	54.2	27.5
Parents average SD	4.9	24.5	26.5	21.0	8.7
Proband average SD	10.1	14.9	29.0	35.2	32.5

Read depth metrics were generated using GATK's DepthOfCoverage tool with "--includeDeletions --countType COUNT_READS --minMappingQuality 20 --minBaseQuality 20 --interval_merging OVERLAPPING_ONLY". Mitochondrial DNA, unlocalized and unplaced contigs were excluded from the analysis.

Table S4. Gene variants shortlisted in each patient

Trio	Gene	cDNA variant	Protein variant	Phase	Genotype	Variant class	ClinVar accession
RaPS_01	*POLE1*	c.5912A>G	p.N1971S	I	CH	4	SCV000778566
		c.5867A>T	p.E1956V			4	SCV000778567
RaPS_02	*COL3A1*	c.2194G>A	p.G732R	I	*de novo*	4	SCV000778568
RaPS_05	*CHD7*	c.3106C>T	p.R1036*	I	*de novo*	5	SCV000778569
RaPS_07	*PIGT*	c.244G>A, c.382G>A, c.550G>A	p.E128K, p.E184K, p.E82K	I	homozygous	4	SCV000778570
RaPS_08	*IL2RG*	c.665G>C	p.R222P	I	XLR	4	
RaPS_11	*WT1*	c.1339G>T, c.1390G>T,	p.D235Y, p.D252Y,	I	*de novo*	4	SCV000778571
		c.703G>T, c.754G>T	p.D447Y, p.D464Y			4	
RaPS_12	*GLDC*	c.2489C>T	p.T830M	I	homozygous	4	SCV000778572
RaPS_15	*RRM2B*	c.887T>G, c.515T>G, c.671T>G	p.I296S, p.I172S, p.I224S	I	CH	5	SCV000778573
		c.121C>T, c.49-6198C>T, c.337C>T	p.R41W, p.R113W			5	SCV000778574
RaPS_16	*NSD1*	c.1066G>T, c.1873G>T	p.G625*, p.G356*	I	*de novo*	5	SCV000778575
RaPS_18	*BCHE*	c.2T>C	p.M1T	II	homozygous	4	SCV000778578
RaPS_21	*TBCE*	c.155_166delGCCACGAAGGGA	p.S52_G55del	I	homozygous	4	SCV000778576
RaPS_24	*CC2D2A*	c.585_586dupTA	p.T196fs*63	I	CH	5	SCV000778577
		inversion (Chr4:15510661–15514794; hg19)				4	

The table lists details of those gene variants identified in this study as likely causative or clinically relevant to feedback. The Phase column refers to the variant analysis Phase through which the variant was identied. The Variant class column refers to the variant classification based on the ACMG guidelines. Classes 4 and 5 refer to likely pathogenic and pathogenic variants, respectively. We have deposited full details of each of our variants to the ClinVar database and provide a link as shown in the ClinVar accession column. CH, compound heterozygous; XLR, X-linked recessive.
